# Formation of a Bacteriostatic Surface on ZrNb Alloy via Anodization in a Solution Containing Cu Nanoparticles

**DOI:** 10.3390/ma13183913

**Published:** 2020-09-04

**Authors:** Viktoriia Korniienko, Oleksandr Oleshko, Yevheniia Husak, Volodymyr Deineka, Viktoriia Holubnycha, Oleg Mishchenko, Alicja Kazek-Kęsik, Agata Jakóbik-Kolon, Roman Pshenychnyi, Katarzyna Leśniak-Ziółkowska, Oksana Kalinkevich, Aleksei Kalinkevich, Marcin Pisarek, Wojciech Simka, Maksym Pogorielov

**Affiliations:** 1 Medical Institute, Sumy State University, 40018 Sumy, Ukraine; vicorn77g@gmail.com (V.K.); oleshkosanya007@gmail.com (O.O.); evgenia.husak@gmail.com (Y.H.); vovadeineka@gmail.com (V.D.); golubnichiy@ukr.net (V.H.); pshenychnyi@gmail.com (R.P.); 2 NanoPrime, 39-200 Dębica, Poland; dr.mischenko@icloud.com; 3 Faculty of Chemistry, Silesian University of Technology, 44-100 Gliwice, Poland; alicja.kazek-kesik@polsl.pl (A.K.-K.); agata.jakobik-kolon@polsl.pl (A.J.-K.); katarzyna.lesniak-ziolkowska@polsl.pl (K.L.-Z.); 4 Institute of Applied Physics NASU, 40000 Sumy, Ukraine; oksana.kalinkevich@gmail.com (O.K.); kalinkevich@gmail.com (A.K.); 5 Institute of Physical Chemistry PAS, 01-224 Warsaw, Poland; mpisarek@ichf.edu.pl

**Keywords:** plasma electrolytic oxidation, dental implant, zirconium-niobium alloy, biocompatibility, bacterial adhesion

## Abstract

High strength, excellent corrosion resistance, high biocompatibility, osseointegration ability, and low bacteria adhesion are critical properties of metal implants. Additionally, the implant surface plays a critical role as the cell and bacteria host, and the development of a simultaneously antibacterial and biocompatible implant is still a crucial challenge. Copper nanoparticles (CuNPs) could be a promising alternative to silver in antibacterial surface engineering due to low cell toxicity. In our study, we assessed the biocompatibility and antibacterial properties of a PEO (plasma electrolytic oxidation) coating incorporated with CuNPs (Cu nanoparticles). The structural and chemical parameters of the CuNP and PEO coating were studied with TEM/SEM (Transmission Electron Microscopy/Scanning Electron Microscopy), EDX (Energy-Dispersive X-ray Dpectroscopy), and XRD (X-ray Diffraction) methods. Cell toxicity and bacteria adhesion tests were used to prove the surface safety and antibacterial properties. We can conclude that PEO on a ZrNb alloy in Ca–P solution with CuNPs formed a stable ceramic layer incorporated with Cu nanoparticles. The new surface provided better osteoblast adhesion in all time-points compared with the nontreated metal and showed medium grade antibacterial activities. PEO at 450 V provided better antibacterial properties that are recommended for further investigation.

## 1. Introduction

Titanium (Ti) dental implants have been widely used in the last 50 years to fix and support prosthetic superstructures from single crowns to fixed and removable prostheses [1]. Despite new commercial implant systems characterized by enhanced surface and mechanical properties, the number of complications is still being increased and varies between 4.5% to 10% [2]. Bacterial infections, implant destruction, abnormal physical activity of the patient, and exhaustion at the tissue-implant boundary are the most common reasons for implantation failure [3]. As reported by a different review, mechanical parameters and surface properties are the main factors responsible for implant success [4].

Since Ti was discovered by William Gregor, it has remained the material of choice for dental implants due to its excellent properties. However, Ti alloys demonstrate a stiffness mismatch with the adjacent bone, which causes a stress shielding effect that leads to bone resorption and implant failure [5]. New metastable β-type Ti alloys have been reported to have the lowest elastic modulus compared to the other Ti alloys. Addition of nontoxic metallic elements such as Nb, Mo, Zr, Sn, and Ta has been widely studied to achieve biomechanical success and high biocompatibility [6]. Several current investigations are focused on zirconium as a very attractive alternative substitute to titanium in dental implantology [7]. It was reported that zirconium–niobium (Zr–Nb) alloys possessed better corrosion resistance and similar biocompatibility compared to Ti–6Al–4V and pure Ti [8,9]. Moreover, some new studies have shown that bacteria attached to zirconium implants poorly compared to titanium ones [10].

In addition to mechanical properties, surface topography is a critical issue for the stimulation of cell proliferation and acceleration of the osseointegration process as well as prevention of bacteria adhesion [11,12,13]. Various surface modifications have been developed for market-available implants using subtractive and additive methods such as grit-blasting, acid etching, electrochemical anodic oxidation, or calcium-phosphate coatings [14]. The plasma electrolytic oxidation (PEO) treatment of dental and orthopedics implants is one of the most promising techniques due to the formation of strong bonds with metallic substrates [15]. There is extensive evidence of the PEO surface’s effectiveness on Ti alloys, demonstrating increasing cell response and better osseointegration compared to the conventional implants [16,17,18]. There are a few studies about Zr-containing PEO implant coatings with successful outcomes [15], but there is lack of information about PEO-treatment of low-modulus ZrNb alloys for dental applications.

PEO coatings are favorable for bacteria due to similar mechanisms of prokaryotic and eukaryotic cell adhesion. Recent studies have demonstrated that metallic nanoparticles (NPs) possess a strong and rapid bactericidal effect due to the production of reactive oxygen species (ROS) as well as causing cell membrane and DNA damage [19,20]. However, R. Pokrowiecki indicated that silver NPs demonstrated cytotoxicity on osteoblasts [21], which requires new safe and biocompatible NPs. Copper NPs are toxic to bacteria because they release Cu^2+^ ions and possess thiophilicity [22]. These properties indicate that CuNPs could be a promising alternative to silver. The PEO method has been already used to produce dental implant Ag-doped surfaces on Ti substrates [23], but there is no information about CuNPs applied for ZrNb implant treatment. Substantial evidence is needed to estimate the antimicrobial properties and biocompatibility of the ZrNb alloy treated with PEO.

## 2. Materials and Methods

### 2.1. Chemicals

The alloy of the zirconium–niobium system (weight%: Zr—97,5; Nb—2.4–2,5; Hf—0.01; Ni—0.01; Cr—0.02; Ti—0.007; Al—0.008; Pb—0.005) was obtained from NanoPrime (Dębica, Poland) and used in the experiment as received. Cylindrical samples were prepared with a diameter of 6 mm and a height of 6 mm. All chemicals were purchased from Sigma Aldrich (Darmstad, Germany) and used as received: copper(II) sulfate pentahydrate (CuSO_4_·5H_2_O, 99%), poly(N-vinylpyrrolidone) (PVP, Mw = 40,000, K-25), sodium phosphinate monohydrate SPM (Na_2_PO_2_·H_2_O, 95%), ethylene glycol (EG) (C_2_H_6_O_2_, 90%), and isopropanol (C_3_H_8_O, 90%).

### 2.2. Synthesis of Cu Nanocrystals

The synthesis of copper nanoparticles was performed in aqueous solution in air at room temperature. At first, 4 g of gum acacia was dissolved in 120 mL of distilled water. The dissolution process lasted nearly 30 min with vigorous stirring of the solution in a 600 mL beaker using a heated magnetic stirrer. Gum acacia was used as a protective medium to prevent copper nanoparticles from sticking and oxidizing after the addition of a reducing agent. A solution of 2.5 g (0.01 mol) of CuSO_4_·5H_2_O in 80 mL of water was prepared in another beaker. Then, a solution of copper salt was added to the cooled solution of gum acacia and stirred continuously and vigorously for 10 min. Then, 3.6 mL of N_2_H_4_·H_2_O was added dropwise to the resulting solution. This volume of reducing agent corresponded to a molar ratio to the used salt of 1:10 [24]. The mixture was stirred for 1 h after the addition of hydrazine hydrate.

**Figure 1:**
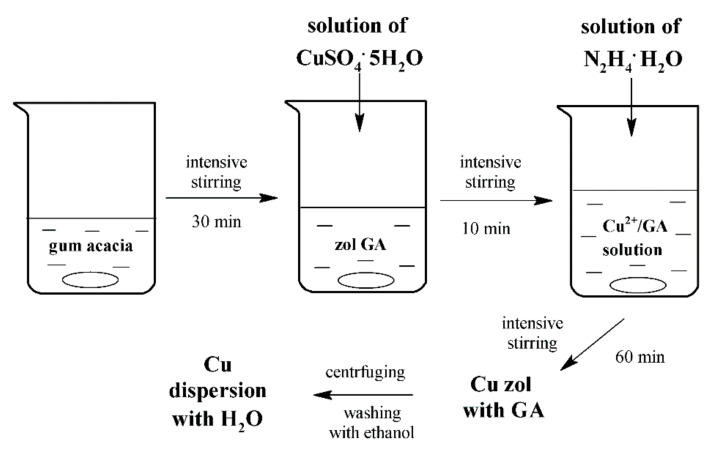
Scheme of synthesis of Cu nanocrystals.

The resulting sol was centrifuged for 20 min with a speed of 5000 rpm. The separated product was washed three times from the organic component with ethanol, followed by centrifugation. The resulting precipitate was dispersed in 100 mL of distilled water using an ultrasonic dispersant. The synthesis scheme is presented in Figure 1.

### 2.3. Nanoparticle Characterization

The microstructural studies to observe the morphology of the individual nanoparticles and obtained material were done using a transmission electron microscope (TEM-125K) (SELMI, Sumy, Ukraine). X-ray diffraction patterns of the samples were recorded using the diffractometer DRON4-07 (“Burevestnik”, St.Petersburg, Russia) with a conventional Bragg-Brentano θ-2θ geometry (where 2θ is Bragg’s angle) equipped with the computer-aided experiment control and data processing system.

### 2.4. Plasma Electrolytic Oxidation (PEO)

All samples were rinsed with distilled water and ultrasonically cleaned in deionized water and 2-propanol for 5 min each. The pretreated samples were subjected to anodization in an electrolytic bath containing Ca(H_2_PO_2_)_2_ (0.5 M), KOH, and copper nanoparticles (5 g/L). The anodic oxidation was performed under a constant current of 0.1 A/cm^2^ and up to final voltages of either 300, 450, and 500 V for 5 min (sample legends: ZrNb-300, ZrNb-450, and ZrNb-500). A DC power supply (PWR 800H, Kikusui, Japan) was used throughout these treatments [25]. The process was performed in a water-cooled electrolysis cell with a titanium mesh cathode and a magnetic stirrer. The ZrNb alloy served as the anode. The surface area of the face that was modified was 1.89 cm^2^ (diameter = 6 mm). The anodized specimens were rinsed with distilled water and deionized water for 5 min. The number of produced samples were enough for each analytical method in at least three replications.

### 2.5. Surface Analysis

Surface topography and chemical structure after the PEO samples were assessed using Tescan (Tescan, Brno-Kohoutovice, Czech Republic) and Phenom Pro X (ThermoFisher, Eindhoven, The Netherlands) scanning electron microscopes with an EDX system X-MaxN20 (Oxford Instruments, Abingdon, UK).

Contact angle (CA) measurement experiments were made using a video-based optical contact angle measuring instrument (OCA 15 EC, Data Physics, Filderstadt, Germany). The CA data were recorded for ultra-pure water for at least three parallel samples.

X-ray diffraction patterns of the samples were recorded using the diffractometer DRON4-07 (“Burevestnik”, St.Petersburg, Russia) with a conventional Bragg-Brentano θ-2θ geometry (where 2θ is Bragg’s angle) equipped with a computer-aided experiment control and data processing system. The Ni-filtered CuKα radiation (wavelength 0.154 nm) was used. The current and the voltage of the X-ray tube were 20 mA and 30 kV, respectively. The sample holder rotated constantly to average the signal from its surface. The patterns were recorded in the continuous registration mode with the rate of 1.0°/min within the 2θ-angle ranging from 10 to 100°. The data processing procedures were carried out with the use of the program package DIFWIN-1 (“Etalon PTC” Ltd., Moscow, Russia). Phase analysis was carried out by comparing the diffraction patterns from the investigated samples and the reference data of JCPDS (Joint Committee on Powder Diffraction Standards).

The chemical composition and chemical state of the prepared sample surface were characterized by X-ray photoelectron spectroscopy (XPS). For this purpose, the PHI 5000 VersaProbe (ULVAC-PHI, Chigasaki, Japan) spectrometer was used. The XPS spectra were excited using AlKα (*hv* = 1486.6 eV, 25 W) monochromatic radiation as a source. The survey and high resolution spectra were collected with the hemispherical analyzer at constant pass energies of 117.4 and 23.5 eV, respectively. The background was corrected using the Smart model to obtain the XPS signal intensity. An asymmetric Gaussian/Lorentzian function at a constant ratio G/L = 0.35 was used for the deconvolution procedure. The determined peak positions were corrected in relation to adventitious carbon C1s at 284.5 eV. Avantage Surface Chemical Analysis software, ThermoFisher Scientific (ver. 5.9911) was used for the data processing.

### 2.6. Analysis of Ion Release from Oxide Coatings

The contents of copper, phosphorus, calcium, and titanium in saline Ringer solutions were determined using the inductively coupled plasma (ICP) atomic emission spectrometer Varian 710-ES (Santa Clara, CA, USA) equipped with the OneNeb nebulizer and twister glass spray chamber. The following parameters were applied: RF power 1.0 kW, plasma flow (argon) 15 L/min, auxiliary flow (argon) 1.5 L/min, nebulizer pressure 210 kPa, pump rate 15 rpm, emission lines of Cu: λ = 327.395 and 324.754 nm, and 206.200 nm, P: λ = 177.434 and 213.618 nm, Ca: λ = 370.602 and 373.690 nm. The calibration curve method was applied. The standards were prepared on a matrix identical to that of the samples (Ringer solution) from single element standard solutions of 1 mg/mL supplied by Merck Millipore (Darmstadt, Germany). Deionized water (Millipore Elix 10 system, Darmstadt, Germany) was used. The results were calculated as the mean value of concentrations obtained for all applied analytical lines for the element concerned with standard deviation not exceeding 1.5%.

### 2.7. Cell Culture

Human primary osteoblasts obtained from patience after informed consent (Institutional Ethical Committee, protocol #2/17 from 14 February 2019) were routinely cultivated in Dulbecco’s Modified Eagle Medium/Nutrient Mixture F-12 (DMEM/F-12) with L-glutamine, containing 100 units/mL penicillin, 100 µg/mL streptomycin, 2.5 µg/mL amphotericin B, 10% Fetal Bovine Serum, and 1.0 ng/mL bFGF. The cells were grown at standard culture conditions of 5% humidified CO_2_ in air at 37 °C with medium renewal every 2–3 days. When the confluence of cells reached 80–85%, osteoblasts were seeded on each sample and positive control wells at a cell density of 2 × 104 cells per well. Nontreated ZrNb alloy was used as a negative control. Cell adhesion and proliferation on surface scaffolds was assessed by the Alamar blue colorimetric assay. Resazurin was added in an amount equal to 10% of the volume to each well. After incubation for 4 h at 37 °C in the dark, absorbance was measured using a Multiskan FC (Thermo Fisher Scientific, Thermo Fisher Scientific, Waltham, MA, USA) plate reader at wavelengths of 570 nm and 600 nm. The cells were quantified at different time intervals: 1 day, 3 days, and 7 days. All experiments were repeated three times. The calculation of the percentage of Alamar blue reduction was performed using the equation according to the manufacturer’s protocol.

### 2.8. Bacteria Adhesion

The isolate of referent strain *S. aureus* was used to study the ability of bacteria to attach to the surface. Initially, microorganisms were cultivated overnight on nutrient broth at 37 °C, and then the suspension of germs in a saline solution was prepared. Microorganisms were mixed with medium, and the final concentration of microorganisms was equal to 1 × 10^5^ Colony-Forming Unit (CFU).

The samples of PEO-coated alloys (including nontreated ZrNb alloy and PEO samples with no Cu NPs) were placed into 2.0 mL of the nutrient broth with the bacterial suspension (Figure 1). After this, the sample was incubated in a plastic 24-well plate at 37 °C for 2, 4, 6, and 24 h. Following this, the disks were taken and washed with sterile 0.9% NaCl solution three times to remove the non-adhered bacteria. Then, the samples were sonicated in the tubes with 1.0 mL of sterile saline solution for 1 min by using an ultrasonic-bath (B3500S-MT, Bransone Ultrasonics Co., Shanghai, China) to separate bacteria that were attached to the surfaces of the specimens. Finally, 10 μL of inoculation aliquots of saline solution were placed onto nutrient agar to count the amount of bacteria by using the streak plate technique. There were negative (media) and positive (bacteria) controls. The untreated ZrNb alloy sample was utilized as a control with a polished untreated surface.

### 2.9. Statistics

The statistical analysis was based on one-way analysis of variance (GraphPad Prism 8.0 software), and a p value of less than 0.05 was considered as statistically significant. All tests were tripled for each time point. The results are expressed as the mean ± standard deviation.

## 3. Results and Discussion

### 3.1. CuNPs Characterization

**Figure 2:**
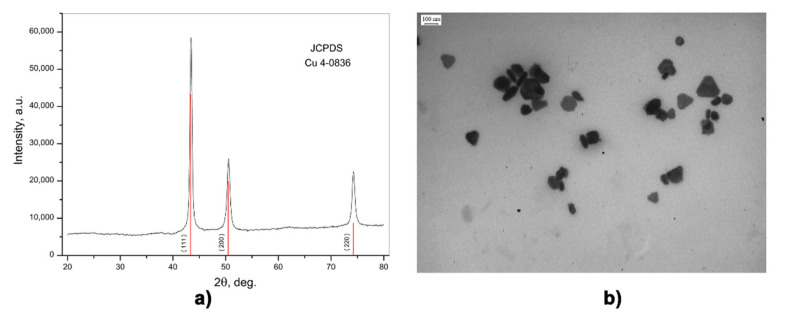
Diffraction pattern (**a**) and TEM image (**b**) of Cu nanocrystals.

Three reflections were observed in the diffractogram of the synthesized substance at the angles 43.431°, 50.553°, and 74.264°, which correspond to reflections from the (111), (200), and (220) crystallographic planes of cubic phase Cu. Extraneous phases were not detected diffractometrically in the material. The characteristics of the diffraction peaks of the formed sample corresponded to JCPDS no. 04-0836 (Figure 2a). The average size of the regions of coherent scattering of the synthesized material was 15.1 nm, calculated by the physical broadening of the diffraction peak (200) using the Scherrer formula:

(1) $$L=\frac{0.94\times \lambda }{\beta \cdot \cos(\theta )}$$

where *λ* is the wavelength of X-ray diffraction; *β* is the value of the physical broadening of the corresponding diffraction peak; and *θ* is the diffraction angle.

The calculated parameters of the unit cell of the synthesized copper corresponded to a = 3.6114 Å, and a volume of V_unit_(Cu) = 47.101 Å^3^. Analysis of the TEM images showed that the synthesized copper nanocrystals had different shapes with the sizes of individual crystals in the range of 10–100 nm (Figure 2b).

### 3.2. Structural Characterization

**Figure 3:**
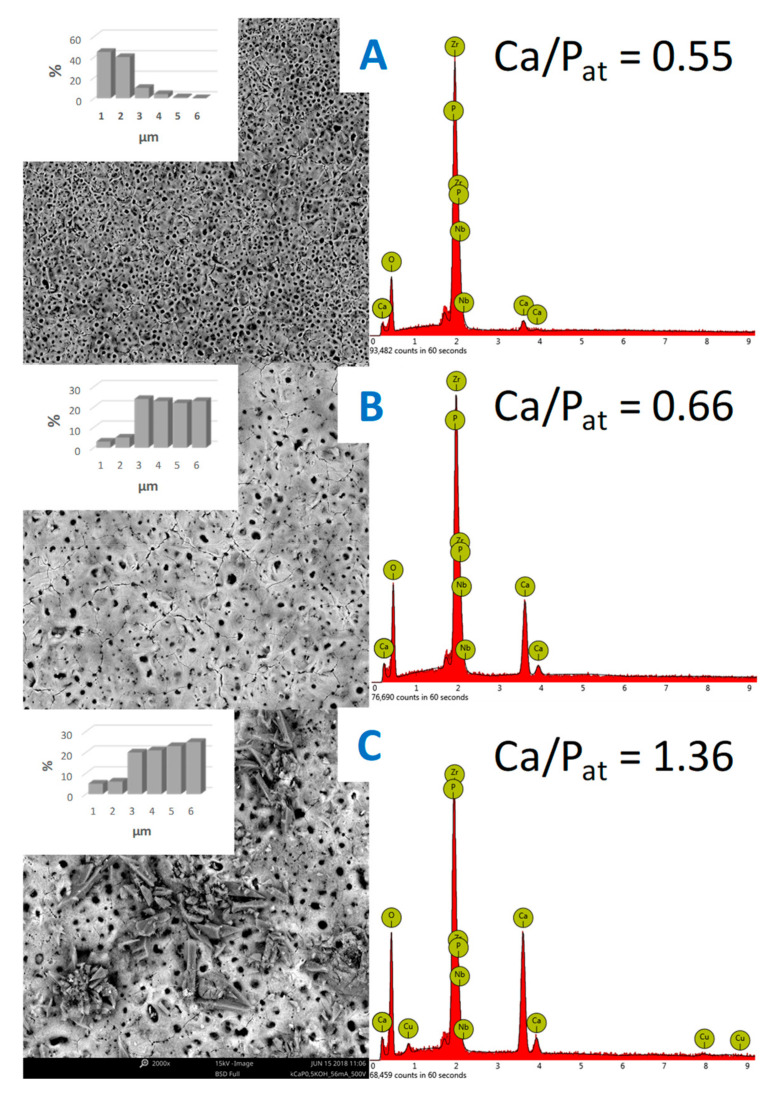
SEM images of the ZrNb alloy surface after PEO in a Cu-containing solution in different modes (300 V—(**A**), 450 V—(**B**), and 500 V—(**C**)) with pore distributions and EDX spectra.

After plasma electrolytic oxidation, the surface of ZrNb acquired a polymorphic character. Longitudinal tubercles with irregular shapes were observed, whose transverse dimensions varied from 5.2 ± 0.7 to 10.4 ± 1.2 micrometers. These structures rose above the overall surface and were accommodated chaotically throughout the area of the samples. In this case, the entire surface of the modified alloy was pierced by cracks (Figure 3) of different lengths and irregular shapes. It is important to point out the appearance of oval pores (craters) of different sizes. Therefore, when using a mode with an electric voltage of 300 V, the internal pore sizes ranged from 1 to 2 microns, with a small number of pores from 3 to 6 micrometers. An increase in voltage up to 450 or 500 V led to the expansion of the craters. The internal dimensions varied from 2.1 ± 0.4 to 6.3 ± 0.8 μm. It should also be noted that when using a mode with a voltage of 500 V, probable phosphate deposits appeared on the surface of the samples (Figure 3C). The transverse dimensions of individual elements were 2–10 micrometers. It is commonly known that the presence of phosphates in the human body affects a wide range of processes. Among them, a bone mineralization process is worth paying attention to. During mineralization, a proper bone tissue structure is being formed. The level of inorganic phosphates strongly influences osteoblast activity and accomplishment of bone mineralization [26]. Thus, the modification of the surface of ZrNb alloys by PEO in a combined solution of copper nanoparticles leads to the formation of mesoporous structures, which should promote cell adhesion on the surface of the samples.

Table 1 presents the water contact angle results. All the investigated ZrNb surfaces after PEO modification were characterized by a contact angle lower than 90°, so it can be concluded that modified surfaces are hydrophilic. Furthermore, the porous structure of the implant material can result in the better wettability of the biomaterial surface. It has been reported that pores existing on the PEO layer support the lower water contact angle and higher wettability due to the possibility of spreading the water particles into the pores [27]. In a different paper [25,28], the decrease in contact angle after PEO treatment of titanium alloy was analogously observed.

Based on EDX analysis, it can be concluded that with increasing voltage, the amount of zirconium in the surface layer decreases whereas phosphorus and calcium amounts are observed to increase. The Zr/Ca ratio decreased from 15.56 in ZrNb-300 to 0.7 in the ZrNb-500 samples, Zr/P simultaneously decreased from 7.51 to 1.0. The Ca/P ratio increased from 0.55 in ZrNb-300 to 1.36 in ZrNb-500. It is worth noting that a Ca/P ratio of 1.36 (ZrNb-500) is strongly similar to the Ca/P ratio for hydroxyapatite (HA), which is equal to 1.67 [29]. Hydroxyapatite is an inorganic calcium phosphate and a main component of human bone tissue. HA is responsible for proper bond formation between the implant material and human bone tissue [30]. This is a reason why a Ca/P ratio of the ZrNb-500 PEO coating similar to HA can result in improvement of the biocompatibility. These data may suggest ceramic coating growth with increased voltage as well as increased incorporation of elements from the anodizing bath to form the oxide layer. The first appearance of copper in the surface layer was detected after the PEO was performed in the electrolyte at 500 V. In some of our previous works, analogous conclusions can be made. It has been observed that increasing voltage during the PEO process of titanium alloy (anodizing bath: 0.1 M Ca(H_2_PO_2_)_2_) results in lowering the Ti/Ca and Ti/P ratios [31].

**Figure 4:**
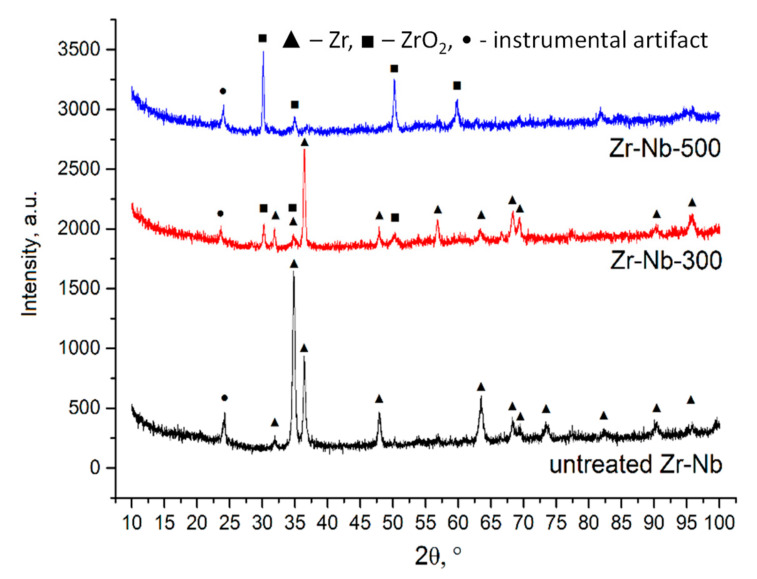
X-ray diffraction patterns of ZrNb alloys with PEO coatings formed in the presence of Cu nanoparticles. For comparison, diffractograms have been stacked.

In Figure 4, the X-ray diffraction patterns of the ZrNb alloy processed with PEO in the electrolyte containing Ca, P, KOH, and Cu nanoparticles are shown. The XRD pattern of the unprocessed alloy is below. The peaks marked with triangles ▲ corresponded to Zr (JCPDS card 5-665). The Nb admixture did not change peak positions in the pattern. The relative intensity of the reflections differed from those of the JCPDS card. In the XRD pattern of the PEO-treated samples at 300 V, a phase of zirconium oxide emerged (ZrO_2_, JCPDS card 49-1642, peaks marked with squares ■). Remarkably, the diffraction pattern of the Zr in this case fully matched the card reference, which may indicate not only the formation of the oxide on the surface, but also the rearrangement the metallic layers. In the pattern of the sample treated at 500 V, only the peaks corresponding to zirconium oxide were visible, which indicates the formation of a thick oxide layer on the surface with a crystal structure of ZrO_2_. It should be noted that no crystal phases containing copper were found. The peak marked with • cannot be ascribed to any crystal phase and is most likely an instrumental artifact.

**Figure 5:**
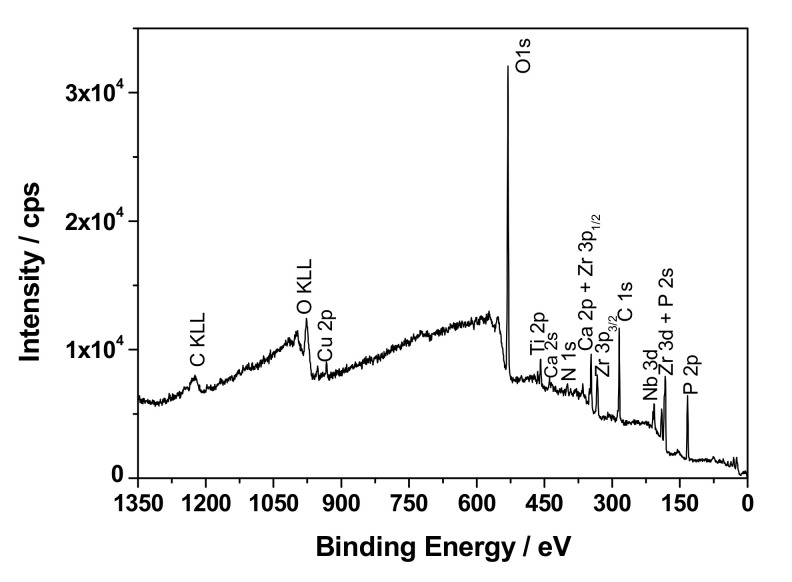
The XPS survey spectrum of the ZrNb-500 sample.

The exemplary coating formed on the ZrNb alloy at 500 V was analyzed using an XPS technique, and the results of the measurements are presented in Figure 5 and Table 1.

**Figure 6:**
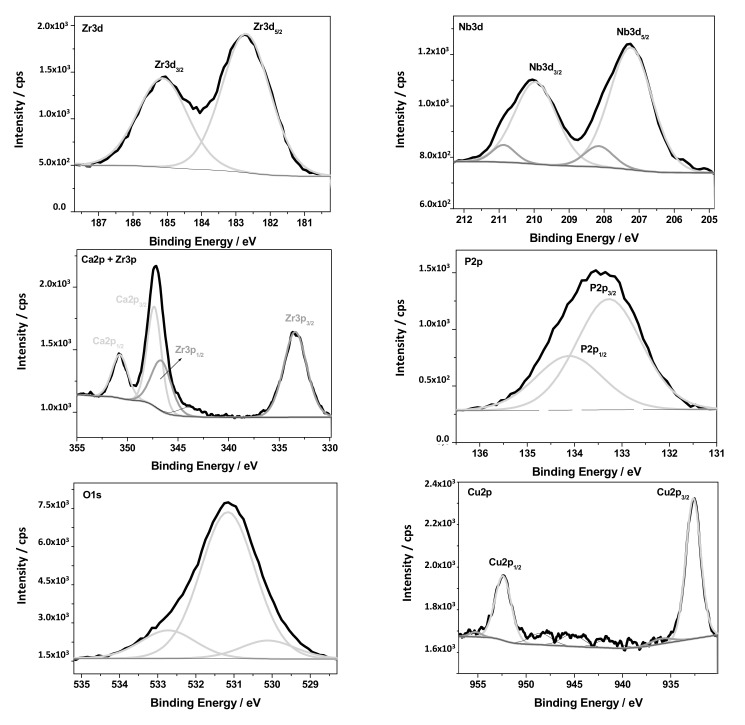
The high resolution XPS spectra after the deconvolution procedure.

The chemical composition of the PEO layer was estimated by XPS spectroscopy. The surface analytical method confirmed the presence of the following elements: O, Zr, Nb, Ti, Ca, P, N, Cu, and C, which are shown in Figure 5. The detailed analysis of the high resolution XPS spectra after the deconvolution procedure revealed that all metallic elements were in oxidized states (Figure 6). The following oxides were found: Nb_2_O_5_ (Nb3d_5/2_—207.2 eV), ZrO—(Zr3d_5/2_—182.7 eV), TiO_2_—(Ti2p_3/2_—458.7 eV), and Cu_2_O—(Cu2p_3/2_—932.7 eV), where the O1s signal at 530.1 was assigned to metal–oxygen bonds. Metal oxides after PEO functionalization of the Zr–Nb alloy in Ca and P-based solutions were detected in the presence of phosphate and carbonate groups. The characteristic signals for O1s at—531.2 eV, P2p_3/2_ at—133.3 eV, Ca2p_3/2_ at—347.4 eV, and C1s at—88.9 eV were attributed to these chemical states. Moreover, a detailed XPS analysis of C1s, O1s, and N1s peaks showed some contribution in the oxide layer of typical impurities in the form of C–C, C–O, C=O, and C–N bonds. No potassium was detected in the analyzed oxide layer. Titanium is probably a contamination after the polishing process. The XPS results are summarized in Table 2 and Figure 6.

### 3.3. Analysis of Ion Release

**Figure 7:**
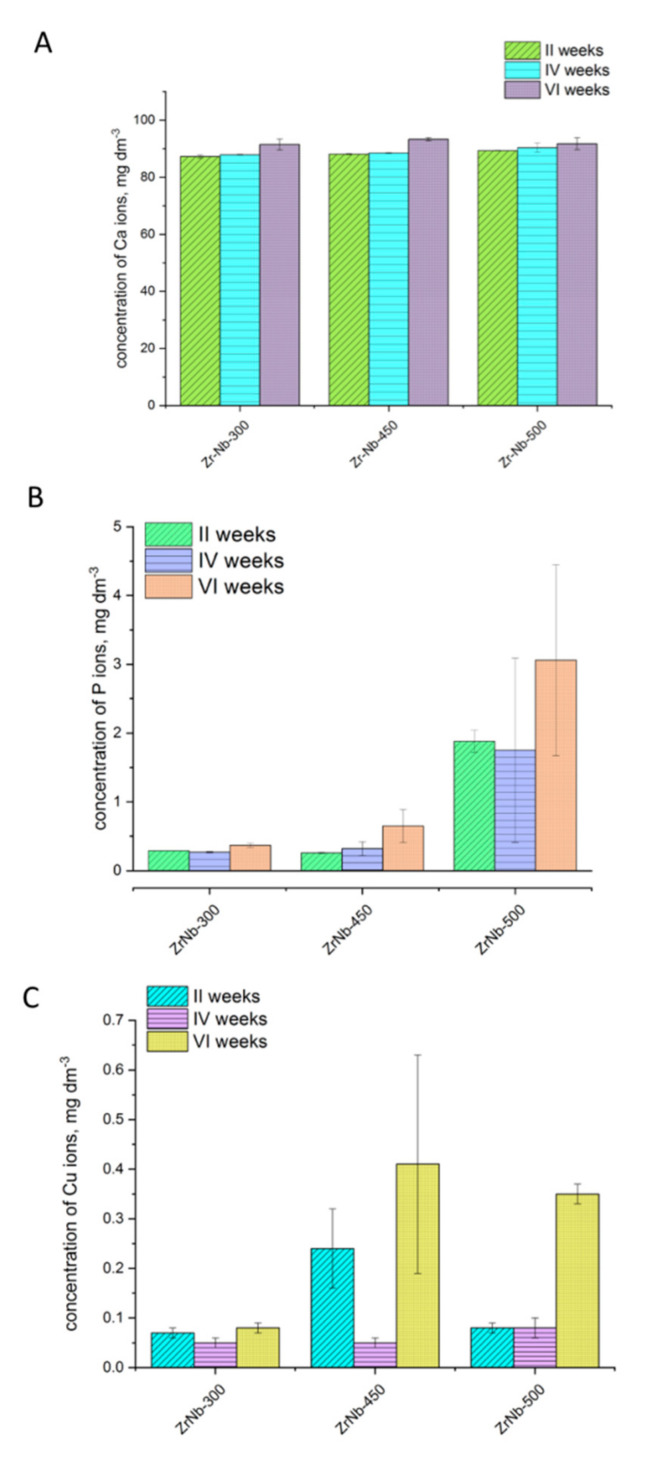
Concentration of the selected ions ((**A**)—Ca; (**B**)—P; (**C**)—Cu) released from the coatings after immersion in Ringer solutions for up to six weeks. Concentration of Zr and Nb ions for all samples after each measurement was below 0.01 mg/L (including the reference sample).

The results of the ions released from the analyzed coatings are presented in Figure 7.

Analyzing the results of different ion release from the PEO coatings, it can be concluded that the concentration of Ca ions was quite stable. For all samples and times of release, the level of Ca concentration was ~80.00–90.00 mg/L, and was not variable within 2–6 weeks of release. A different conclusion can be made in the case of phosphorus ion concentration. The P ion concentrations for ZrNb-300 and ZrNb-450 were not higher than 1.00 mg/L, even after six weeks of release. However, the ZrNb surface anodized in 500 V was characterized by the higher P ion release, approximately 0.35–0.40 mg/L. This can be related to the higher phosphorus incorporation into the oxide layer during PEO because of the higher voltage (500 V) application. Similar results were obtained for Cu ion release. The samples anodized in lower voltage (300 V) are characterized with a Cu ion release lower than 0.10 mg/L, while the treatments using 450 V and 500 V voltage followed with Cu ion release concentrations of ~0.35–0.40 mg/L.

Although the XRD analysis did not confirm the presence of copper, the EDX, XPS, and ICP analysis did. This can be explained by the presence of a small amount of copper in the oxide layer. Additionally, the copper incorporated into the PEO coating can be amorphous.

### 3.4. Cell Culture

**Figure 8:**
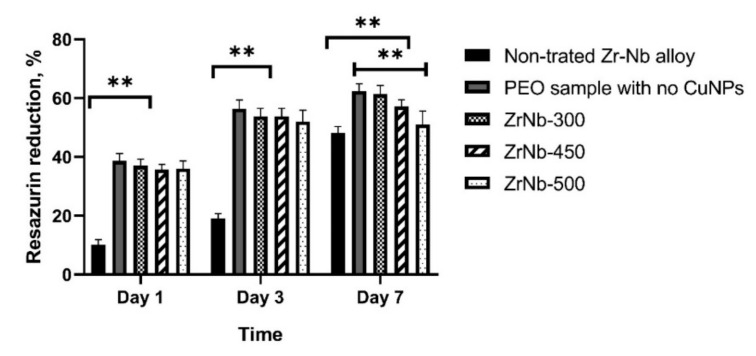
The resazurin reduction assay test at different times of osteoblast cultivation in ZrNb samples with PEO in different voltage modes. Asterisks indicate the *p* value between groups: **—*p* < 0.001.

Figure 8 presents the results of the cytocompatibility of the samples before and after PEO treatment (with CuNP addition). After 24 h and three days of cells proliferation, the untreated, via the PEO surface, revealed significantly lower cytocompatibility; the resazurin percent of reduction was lower than 20%, while it was ~35–63% for the PEO oxide coatings with incorporated CuNPs. A different observation can be made for samples after seven days of cell incubation. In this case, the untreated implant surface was also characterized by relatively high cytocompatibility where its resazurin percent of reduction was ~55%. Although all the investigated samples indicated good cytocompatible properties after seven days, it is worth noting that the PEO CuNP-incorporated coating revealed better cytocompatibility already after one day. This is a very important feature to ensure the proper growth and proliferation of living cells right after implantation.

### 3.5. Bacterial Adhesion Test

**Figure 9:**
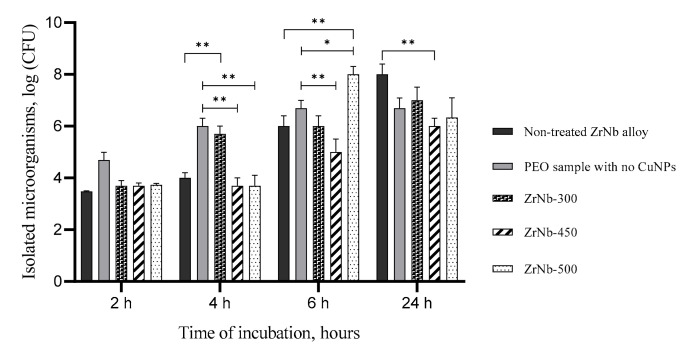
The number of attached bacteria on the surfaces of ZrNb samples at different time points of incubation. The data are mean and SD values. Asterisks indicate the *p* value between groups: *—*p* < 0.005; **—*p* < 0.0001.

All samples with CuNPs demonstrated similar bacteria adhesive properties in 2 h after cocultivation with the maximum *S. Aureus* concentration at 3.7 log CFU (Figure 9).

There were no significant differences between the number of bacteria on the control samples (both nontreated Zr–Nb alloy and PEO samples with no CuNPs) and ZrNb-300, ZrNb-450, and ZrNb-500 ones. Excessive bacteria growth was detected on the PEO control and ZrNb-300 samples after 4 h of incubation (up to 6 log CFU) with significant differences compared to the ZrNb-450 and ZrNb-500 ones (*p* < 0.0001) that provided antiadhesive activity. However, we could see rapid bacteria growth on the ZrNb-500 surface at 6 h compared to all other samples (*p* < 0.0001). It should be noted that all PEO-treated samples showed less bacteria adhesion in 24 h of cocultivation compared to the nontreated ones, but significant differences could only be found in the ZrNb-450 regimen. The lowest bacteria adhesion on the ZrNb-450 surface may be related to the highest Cu ion release from the ZrNb-450 surface determined by the ICP analysis (Figure 7). The antibacterial properties of PEO Cu-doped PEO coatings were not better during the first 2–6 h in comparison to the untreated surface. Although, after 24 h, all three types of CuNP-incorporated PEO coatings revealed stronger bacteriostatic effects than the reference untreated surface. In this case, the amount of *S. aureus* bacteria was significantly lower, ~10–100 times than for the untreated via the PEO surface. The results indicate that CuNP-incorporated PEO layers obtained on the implant surfaces exhibit bacteriostatic properties within 24 h of the implantation process. The implantation process, as a kind of surgery and intervention in humans, requires protection against bacteria attack for the first few days after surgery, so it is important to ensure strong bacteriostatic properties right after the implantation process that do not decrease.

Plasma electrolytic oxidation has become a method of choice to provide a biocompatible corrosion-resistance ceramic coating for medical implants [32]. There is significant research on Ti-based alloy PEO coatings with high biocompatibility and in vivo effectiveness [18,25]. Some researchers have effectively anodized an alloy containing Zr and Nb, which opens up perspectives for the wide application of PEO for medical implant production [15]. To enhance antibacterial properties of the modified surface, antibiotics [33] and silver nanoparticles [23] have been added to the PEO solution. Despite the antibacterial effect, antibiotics and AgNPs exhibit different levels of toxicity that limited their application for clinical use [21], stimulating the development of new ways to overcome the current limitations. Due to their low toxicity, copper nanoparticles may be an alternative to silver in the development of antibacterial surfaces in medical implants. Our recent research shows the safety and high effectiveness of CuNPs made using a green synthesis approach against a wide range of pathogens [34], but no research for copper application in PEO coatings has been reported. The current research has shown the formation of a complex mesoporous coating in the ZrNb alloy using a Ca–P solution doped with CuNPs. Voltage control allows thick coatings with low Zr/Ca and Zr/P rates to be obtained with the 450 V mode, similar to other research reported with the formation of a PEO coating without NP addition [35]. We found a strong antibacterial effect of the CuNP-incorporated surface similar to the silver effect [36]. Based on the current data, we can conclude that the main mechanism is the prevention of bacteria adhesion to the PEO coating that allows following cell colonization and the prevention of infectious complications [37]. The main advantages of the new CuNP-incorporated PEO surface is the high biocompatibility. It is known that PEO coatings exhibit high biocompatibility and could stimulate osseous cell attachment and proliferation [38]. The current research proved that CuNPs did not affect cell attachment and proliferation within seven days compared to the conventional surface and exhibited more biocompatibility compared to the other NP-incorporated surface [39]. Taking into account the complex antibacterial effect with the high biocompatibility of the CuNP-doped PEO coating opens up new perspectives for the development of medical implants with advanced antibacterial properties for the prevention of infectious complications.

## 4. Conclusions

Plasma electrolytic oxidation of a ZrNb alloy in a Ca–P solution with CuNPs formed a stable ceramic layer incorporated with Cu nanoparticles. The new surface provided better osteoblast adhesion at all time-points compared to the nontreated metal and showed medium grade antibacterial activities. PEO at 450 V can provide better antibacterial properties that can be recommended for further investigation.

## Figures and Tables

**Table 1:** Water contact angle of ZrNb surfaces after PEO treatment in CuNPs containing baths.

Sample	Contact Angle, °
ZrNb-300	57.4 ± 1.9
ZrNb-450	40.7 ± 4.3
ZrNb-500	28.7 ± 1.4

**Table 2:** The XPS data evaluated from the deconvolution of Nb3d, Zr3d, Ti2p, Cu2p, P2p, Ca2p, O1s, C1s, and N1s XPS spectra recorded for the ZrNb-500 sample.

Binding Energy/eV High Resolution Spectra									Chemical Bonds/States	Chemical Composition/at.%
Nb3d_5/2_	Zr3d_5/2_	Ti2p_3/2_	Cu2p_3/2_	P2p_3/2_	Ca2p_3/2_	O1s	C1s	N1s		
207.2	182.7	458.7	932.7			530.1			Me-O	Nb—1.2
										C—28.4
				133.3	347.4	531.2	288.9		PO_4_^3−^, CO_3_^2−^	Ca—3.0
										P—14.5
										Zr—3.9
							284.5		C-C	N—1.5
							285.7	399.6	C-O/C-N	Ti—2.0
						532.7	287.4		C=O	O—44.7
								401.3	NH_x_	Cu—1.0

## References

[1] Insua A., Monje A., Wang H.L., Miron R.J. (2017). Basis of bone metabolism around dental implants during osseointegration and peri-implant bone loss. J. Biomed. Mater. Res. Part A.

[2] Liaw K., Delfini R.H., Abrahams J.J. (2015). Dental Implant Complications. Semin. Ultrasound CT MRI.

[3] Kirmanidou Y., Sidira M., Drosou M.E., Bennani V., Bakopoulou A., Tsouknidas A., Michailidis N., Michalakis K. (2016). New Ti-Alloys and Surface Modifications to Improve the Mechanical Properties and the Biological Response to Orthopedic and Dental Implants: A Review. BioMed Res. Int..

[4] Al-Radha A.S.D., Dymock D., Younes C., O’Sullivan D. (2012). Surface properties of titanium and zirconia dental implant materials and their effect on bacterial adhesion. J. Dent..

[5] Shirazi H.A., Ayatollahi M.R., Asnafi A. (2017). To reduce the maximum stress and the stress shielding effect around a dental implant–bone interface using radial functionally graded biomaterials. Comput. Methods Biomech. Biomed. Eng..

[6] Yu S.R., Zhang X.P., He Z.M., Liu Y.H., Liu Z.H. (2004). Effects of Ce on the short-term biocompatibility of Ti-Fe-Mo-Mn-Nb-Zr alloy for dental materials. J. Mater. Sci. Mater. Med..

[7] Hashim D., Cionca N., Courvoisier D.S., Mombelli A. (2016). A systematic review of the clinical survival of zirconia implants. Clin. Oral Investig..

[8] Zhao X.L., Li L., Niinomi M., Nakai M., Zhang D.L., Suryanarayana C. (2017). Metastable Zr–Nb alloys for spinal fixation rods with tunable Young’s modulus and low magnetic resonance susceptibility. Acta Biomater..

[9] Mishchenko O., Ovchynnykov O., Kapustian O., Pogorielov M. (2020). New Zr-Ti-Nb alloy for medical application: Development, chemical and mechanical properties, and biocompatibility. Materials.

[10] Hao Y., Huang X., Zhou X., Li M., Ren B., Peng X., Cheng L. (2018). Influence of dental prosthesis and restorative materials interface on oral biofilms. Int. J. Mol. Sci..

[11] Patel A.R., Patra F., Shah N.P., Shukla D. (2017). Biological control of mycotoxins by probiotic lactic acid bacteria. Dyn. Dairy Ind. Consum. Demands.

[12] Ehlers M.R., Todd R.M. (2017). Genesis and Maintenance of Attentional Biases: The Role of the Locus Coeruleus-Noradrenaline System. Neural Plast..

[13] Li B., Ge Y., Wu Y., Chen J., Xu H.H., Yang M., Li M., Ren B., Feng M., Weir M.D. (2017). Anti-Bacterial and Microecosystem-Regulating Effects of Dental Implant Coated with Dimethylaminododecyl Methacrylate. Molecules.

[14] Velasco-Ortega E., Ortiz-García I., Jiménez-Guerra A., Monsalve-Guil L., Muñoz-Guzón F., Perez R.A., Gil F.J. (2019). Comparison between sandblasted acid-etched and oxidized titanium dental implants: In vivo study. Int. J. Mol. Sci..

[15] Michalska J., Sowa M., Piotrowska M., Widziołek M., Tylko G., Dercz G., Socha R.P., Osyczka A.M., Simka W. (2019). Incorporation of Ca ions into anodic oxide coatings on the Ti-13Nb-13Zr alloy by plasma electrolytic oxidation. Mater. Sci. Eng. C.

[16] Polo T.O.B., da Silva W.P., Momesso G.A.C., Lima-Neto T.J., Barbosa S., Cordeiro J.M., Hassumi J.S., da Cruz N.C., Okamoto R., Barão V.A. (2020). Plasma Electrolytic Oxidation as a Feasible Surface Treatment for Biomedical Applications: An in vivo study. Sci. Rep..

[17] Yeung W.K., Reilly G.C., Matthews A., Yerokhin A. (2013). In vitro biological response of plasma electrolytically oxidized and plasma-sprayed hydroxyapatite coatings on Ti-6Al-4V alloy. J. Biomed. Mater. Res. Part B Appl. Biomater..

[18] Chung C.J., Su R.T., Chu H.J., Chen H.T., Tsou H.K., He J.L. (2013). Plasma electrolytic oxidation of titanium and improvement in osseointegration. J. Biomed. Mater. Res. Part B Appl. Biomater..

[19] Siddiqi K.S., Husen A., Rao R.A.K. (2018). A review on biosynthesis of silver nanoparticles and their biocidal properties. J. Nanobiotechnol..

[20] Escárcega-González C.E., Garza-Cervantes J.A., Vazquez-Rodríguez A., Montelongo-Peralta L.Z., Treviño-Gonzalez M.T., Castro E.D.B., Saucedo-Salazar E.M., Morales R.C., Soto D.R., González F.T. (2018). In vivo antimicrobial activity of silver nanoparticles produced via a green chemistry synthesis using acacia rigidula as a reducing and capping agent. Int. J. Nanomed..

[21] Pokrowiecki R., Zaręba T., Szaraniec B., Pałka K., Mielczarek A., Menaszek E., Tyski S. (2017). In vitro studies of nanosilver-doped titanium implants for oral and maxillofacial surgery. Int. J. Nanomed..

[22] Molteni C., Abicht H.K., Solioz M. (2010). Killing of bacteria by copper surfaces involves dissolved copper. Appl. Environ. Microbiol..

[23] Oleshko O., Deineka V.V., Husak Y., Korniienko V., Mishchenko O., Holubnycha V., Pisarek M., Michalska J., Kazek-Kęsik A., Jakóbik-Kolon A. (2019). Ag nanoparticle-decorated oxide coatings formed via plasma electrolytic oxidation on ZrNb alloy. Materials.

[24] Chawla P., Kumar N., Bains A., Dhull S.B., Kumar M., Kaushik R., Punia S. (2020). Gum arabic capped copper nanoparticles: Synthesis, characterization, and applications. Int. J. Biol. Macromol..

[25] Leśniak-Ziółkowska K., Kazek-Kęsik A., Rokosz K., Raaen S., Stolarczyk A., Krok-Borkowicz M., Pamuła E., Gołda-Cępa M., Brzychczy-Włoch M., Simka W. (2020). Electrochemical modification of the Ti-15Mo alloy surface in solutions containing ZnO and Zn_3_(PO4)_2_ particles. Mater. Sci. Eng. C.

[26] Penido M.G., Alon U.S. (2012). Phosphate homeostasis and its role in bone health. Pediatric Nephrol..

[27] Santos-Coquillat A., Martínez-Campos E., Mohedano M., Martínez-Corriá R., Ramos V., Arrabal R., Matykina E. (2018). In vitro and in vivo evaluation of PEO-modified titanium for bone implant applications. Surf. Coat. Technol..

[28] Kazek-Kęsik A., Kalemba-Rec I., Simka W. (2019). Anodization of a Medical-Grade Ti-6Al-7Nb Alloy in a Ca(H_2_PO_2_)_2_-Hydroxyapatite Suspension. Materials.

[29] Rey C., Combes C., Drouet C., Grossin D. (2011). 1.111–Bioactive Ceramics: Physical Chemistry. Compr. Biomater..

[30] LeGeros R.Z. (2008). Calcium phosphate-based osteoinductive materials. Chem. Rev..

[31] Simka W., Socha R.P., Dercz G., Michalska J., Maciej A., Krząkała A. (2013). Anodic oxidation of Ti–13Nb–13Zr alloy in silicate solutions. Appl. Surf. Sci..

[32] Rafieerad A.R., Ashra M.R., Mahmoodian R., Bushroa A.R. (2015). Surface characterization and corrosion behavior of calcium phosphate-base composite layer on titanium and its alloys via plasma electrolytic oxidation: A review paper. Mater. Sci. Eng. C.

[33] Bigham A., Saudi A., Rafienia M., Rahmati S., Bakhtiyari H., Salahshouri F., Sattary M., Hassanzadeh-Tabrizi S.A. (2019). Electrophoretically deposited mesoporous magnesium silicate with ordered nanopores as an antibiotic-loaded coating on surface-modified titanium. Mater. Sci. Eng. C.

[34] Holubnycha V., Pogorielov M., Korniienko V., Kalinkevych O., Ivashchenko O., Peplinska B., Jarek M. Antibacterial activity of the new copper nanoparticles and Cu NPs/chitosan solution. Proceedings of the IEEE 7th International Conference on Nanomaterials: Applications and Properties, NAP 2017.

[35] Sowa M., Simka W. (2018). Effect of DC plasma electrolytic oxidation on surface characteristics and corrosion resistance of zirconium. Materials.

[36] Zhang K., Wang S., Zhou X., Xu H.H.K., Weir M.D., Ge Y., Li M., Wang S., Li Y., Xu X. (2015). Effect of antibacterial dental adhesive on multispecies biofilms formation. J. Dent. Res..

[37] Grischke J., Eberhard J., Stiesch M. (2016). Antimicrobial dental implant functionalization strategies—A systematic review. Dent. Mater. J..

[38] Smeets R., Stadlinger B., Schwarz F., Beck-Broichsitter B., Jung O., Precht C., Kloss F., Gröbe A., Heiland M., Ebker T. (2016). Impact of Dental Implant Surface Modifications on Osseointegration. BioMed Res. Int..

[39] Necula B.S., van Leeuwen J.P.T.M., Fratila-Apachitei L.E., Zaat S.A.J., Apachitei I., Duszczyk J. (2012). In vitro cytotoxicity evaluation of porous TiO2-Ag antibacterial coatings for human fetal osteoblasts. Acta Biomater..

